# Bioengineered Iron Nanoparticles From *Canavalia cathartica* Seeds: A Promising Nanobiosorbent for Cr (VI) Remediation

**DOI:** 10.1002/cbdv.71847

**Published:** 2026-10-20

**Authors:** Neethu Cyril, James Baben George, Sylas Variyattel Paulose, Laigi Joseph

**Affiliations:** ^1^ Department of Chemistry Assumption College Kottayam Kerala India; ^2^ School of Environmental Sciences Mahatma Gandhi University Kottayam Kerala India; ^3^ Department of Chemistry St. Berchman's College Kottayam Kerala India; ^4^ Department of Chemistry T.M.J.M Government College, Manimalakunnu Oliyapuram P. O, Koothattukulam Kerala India

**Keywords:** adsorption isotherm, antioxidant, *Canavalia cathartica*, chromium removal, iron nanoparticles

## Abstract

This study reports the green synthesis of iron nanoparticles (FeNP‐CC) using aqueous seed extract of *Canavalia cathartica*, a less explored mangrove associate, and evaluates their efficacy in the remediation of hexavalent chromium (Cr(VI)) from aqueous systems. Phytochemical and spectroscopic analyses using LC‐MS/MS, UV–visible spectroscopy, FTIR, HR‐TEM, and XRD confirmed that bioactive constituents such as polyphenols and L‐canavanine acted as both reducing and stabilizing agents during the synthesis of FeNP‐CC, resulting in nanoparticles with a nanocrystalline core–shell morphology. Adsorption experiments revealed that Cr(VI) removal followed pseudo‐second‐order kinetics, suggesting a chemisorption‐dominated mechanism. Equilibrium studies fitted best to the Freundlich isotherm model (*R* ^2^ = 0.99), indicating multilayer adsorption on a heterogeneous surface. Optimal removal (100%) was achieved for 10 mg/L Cr(VI) within 50 min under acidic conditions. Furthermore, FeNP‐CC successfully remediated complex, real‐world laboratory wastewater containing 30 mg/L Cr(VI) despite competitive matrix interferences. Overall, this biogenic nanobiosorbent represents a highly sustainable, low‐cost, and efficient candidate for practical wastewater purification.

## Graphical abstract

Stable, core‐shell iron nanoparticles (FeNP‐CC) were green‐synthesized using *Canavalia cathartica* seeds rich in L‐canavanine, serving as the primary reductant and capping agent. Under optimal acidic conditions (pH 4.0), FeNP‐CC achieved 100% Cr(VI) removal within 50 min. This highly efficient remediation proceeds via sequential electrostatic attraction, inner‐sphere coordinate complexation, core‐mediated zero‐valent iron reduction to Cr(III), and final insoluble surface co‐precipitation.

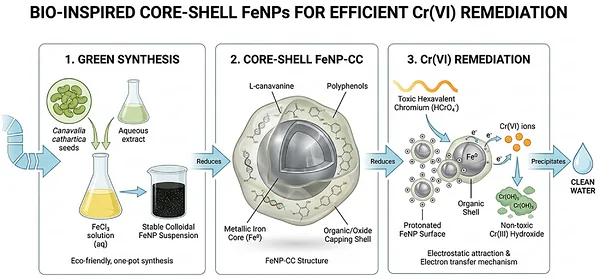

## 1 Introduction

Among various heavy metal pollutants, chromium contamination in aqueous environments remains a significant global environmental concern. Chromium is widely utilized in industrial processes such as electroplating, pigment manufacturing, textile production, leather tanning, and the manufacture of refractory materials [1]. It exists primarily in two stable oxidation states in nature: hexavalent chromium Cr(VI) and trivalent chromium Cr(III). Of these, Cr(VI) is approximately 100 times more toxic than Cr(III) and has been classified as a Group A human carcinogen by the US Environmental Protection Agency (EPA). In aqueous environments, Cr(VI) exhibits high solubility and mobility, which greatly amplifies its hazard potential compared to the relatively immobile and less toxic Cr(III) species [2]. Consequently, remediation technologies capable of reducing toxic Cr(VI) to the stable, benign Cr(III) form are considered highly beneficial and sustainable environmental strategies.

Various conventional methods, including electrochemical precipitation [3], reverse osmosis [4], and ion exchange [5], have been employed for Cr(VI) reduction. However, these methods are often limited by high operational costs and low cost‐effectiveness. Recently, metallic nanoparticles have attracted significant interest for heavy metal remediation due to their exceptional physical and chemical properties, such as enhanced catalytic activity, high biocompatibility, superior adsorption capacity, large surface‐area‐to‐volume ratios, reusability, excellent dispersibility, surface functionalization potential, and cost‐effectiveness [6]. Although physical and chemical methods are widely employed for nanoparticle synthesis, they are generally unfavorable due to high energy consumption, substantial equipment costs, and the generation of hazardous chemical byproducts. In contrast, the green synthesis of iron nanoparticles is highly favored owing to its low cost, eco‐friendly nature, and high efficiency under ambient environmental conditions.

The stabilization of biogenic nanoparticles in aqueous suspensions is fundamentally governed by complex interfacial chemistry; understanding the interfacial dilational rheology and surfactant‐like behavior of capping biomolecules is essential to prevent nanoparticle passivation and maintain active surface sites [7]. This rapidly expanding domain of green nanotechnology has recently demonstrated remarkable success in using diverse biogenic nanomaterials and advanced heterojunctions for the degradation of persistent organic pollutants and hazardous industrial wastewater effluents. For instance, highly active, green synthesized cadmium oxide (CdO) nanoparticles have achieved exceptional degradation of methyl orange dyes [8], and Zn‐doped CuO–CdS nanocomposites were prepared for photocatalytic decomposition of organic pollutants [9]. Biogenic zinc oxide (ZnO), copper oxide (CuO), and bimetallic ZnO/CuO nanocomposites have emerged as powerful platforms for the rapid purification of industrial effluents [10]. Recent advances have also showcased the design of advanced green‐synthesized materials, such as ZnO‐NiO heterojunctions, which facilitate enhanced charge carrier separation for the degradation of complex pharmaceuticals like ciprofloxacin [11].

The biosynthesis of zero‐valent iron nanoparticles requires effective reducing conditions, as the standard reduction potentials for the Fe^3^^+^/Fe^0^ and Fe^2^^+^/Fe^0^ couples are −0.04 and −0.44 V, respectively, indicating the relatively challenging reduction of iron ions to metallic Fe^0^ [12]. To date, various plant extracts—including those from *Globularia trichosantha* [13], Coffee [14], *Pistacia‐atlantica* [15], *Croton macrostachyus* [16], *Camellia sinensis* [17], *Rosa indica* [18], and *Palm Petiole* [19]—have been successfully employed for the synthesis of nanoscale zero‐valent iron (nZVI) with diverse shapes, sizes, and properties, which directly influence their pollutant removal efficiency. For example, iron nanoparticles synthesized using coffee extracts effectively adsorb Cr(VI) at pH 3.0 and 30°C, whereas those prepared using *Globularia trichosantha* flowers remove Cr(VI) at pH 2.0 and 40°C. Similarly, nanoparticles synthesized via *Croton macrostachyus* extract are optimized for Cr(VI) removal at pH 6.0 and 40°C.

Despite these advancements, a critical scientific lacuna remains: many green‐synthesized iron nanoparticles suffer from rapid colloidal aggregation, surface passivation, and low physical stability in aqueous systems, which severely curtails their long‐term active surface area and Cr(VI) removal performance. To address this gap, this study investigates the biosynthesis of highly stable iron nanoparticles (FeNP‐CC) using the aqueous seed extract of *Canavalia cathartica*, a neglected and underutilized species (NUS). As a mangrove associate, *C. cathartica* thrives in hostile coastal environments marked by extreme salinity, high moisture levels, intense winds, and tidal fluctuations. These challenging conditions trigger evolutionary biochemical adaptations, forcing the plant to synthesize and accumulate highly specialized, biologically active secondary metabolites. Crucially, the seeds of *C. cathartica* are exceptionally rich in L‐canavanine [20]—a non‐proteinogenic amino acid structurally analogous to arginine—alongside highly active flavonoids, saponins, and tannins [21]. The unique molecular structure of L‐canavanine features highly reactive guanidino, amino, and carboxyl functional groups that exhibit an extraordinary affinity for metal surfaces, acting as a powerful capping matrix to prevent nanoparticle aggregation while facilitating the rapid reduction of iron ions. By leveraging this underexplored, stress‐adapted phytochemical profile, we present a sustainable and superior route for synthesizing stable nanocrystalline core‐shell iron nanoparticles optimized for the synergistic electrostatic capture and redox reduction of toxic Cr(VI).

## 2 Materials and Methods

### 2.1 Materials

The seeds of *Canavalia cathartica* Thouars utilized for this research were collected on May 25, 2025, from Kumarakom, a region situated within Kuttanad, part of the UNESCO‐designated Vembanad‐Kol wetland ecosystem (Ramsar site) in Kerala, India. Botanical identification was performed by Dr. Pramod Kumar N, Assistant Professor and an expert plant taxonomist at the Department of Botany, NSS College, Ottapalam, Palakkad, Kerala, India. A voucher specimen was subsequently deposited in the Regional Herbarium of Kerala, located within the Department of Botany, St. Berchmans College, Changanacherry, Kerala, under accession number RHK7666 (Supporting Information). Analytical‐grade reagents and chemicals required for this investigation were procured from Merck India Ltd.

### 2.2 Synthesis of *C. cathartica* Stabilized Iron Nanoparticles (FeCC‐NP)

Dried seeds of *C. cathartica* (10 g) were finely powdered and extracted with 50 mL of double‐distilled water for 15 min at 90°C. The resulting supernatant was filtered through Whatman Grade 40 filter paper and stored at 4°C for subsequent experiments. For the biosynthesis of FeNP‐CC, the aqueous seed extract was mixed with a 0.01 M aqueous solution of FeCl_3_ at a volume ratio of 1:2 at room temperature (37°C) under constant stirring at 100 rpm. The synthesized iron nanoparticles were then vacuum freeze‐dried at −45.15°C and stored for further characterization.

### 2.3 Phytochemical Analysis of Aqueous Seed Extract of *C. cathartica*

#### 2.3.1 Qualitative Analysis

The following standard preliminary phytochemical tests were conducted to qualitatively assess the presence of bioactive constituents in the aqueous seed extract of *C. cathartica*: [22]—alkaloids (Dragendorff's reagent test), flavonoids (alkaline reagent test), saponins (froth test), proteins (ninhydrin test), cardiac glycosides (Keller–Kiliani test), and tannins (lead acetate test) (Supporting Information).

#### 2.3.2 Quantitative Analysis

Quantitative spectrophotometric assays were conducted to determine the antioxidant and bioactive profiles of the aqueous seed extract of *C. cathartica*. These analyses comprised the determination of the total phenolic content (TPC), ferric reducing antioxidant power (FRAP) activity, and total antioxidant capacity (TAC) (Supporting Information).

### 2.4 Characterization Techniques

UV‐visible studies were carried out using a Shimadzu UV‐1700 Pharmaspec spectrophotometer. The dried extract and FeCC‐NP were analyzed by FTIR spectroscopy. The IR spectra were recorded using a Shimadzu IR Prestige‐21 FTIR spectrometer equipped with a ZnSe‐ATR crystal. Spectral acquisition was carried out over a scan range of 600–4000 cm^−1^ with 30 scans per sample and a resolution of 4 cm^−1^. Analysis of the extract was carried out using a Q‐TOF LC‐MS system, comprising an Agilent 1290 Infinity Liquid Chromatography unit coupled to a Shimadzu LCMS‐8040 model tandem mass spectrometer equipped with an electrospray ionization (ESI) source operating in both negative and positive ionization modes. Chromatographic separation was achieved using an Agilent ZORBAX SB‐C18 column (2.1 mm × 150 mm, 3.5 µm particle size) maintained at 25°C. The mobile phase consisted of solvent A (0.1% formic acid in distilled water) and solvent B (0.1% formic acid in acetonitrile). The injection volume was set at 1.0 µL, with a flow rate of 0.5 mL/min. The ESI conditions used were an interface temperature of 350°C, a dissolution line temperature of 250°C and 400°C in the ESI source. Nitrogen was used as the nebulizing gas and drying gas at flow rates of 3 and 15 L min^−1^, respectively, and the interface voltage used was 4.50 kV. Data were acquired over an m/z range of 100–1000. Zeta potential measurements were carried out by the Litesizer 500 Anton Paar GmbH analyzer. The size, morphology, and elemental composition of iron nanoparticles were examined by high‐resolution transmission electron microscopy (HRTEM) equipped with EDX measurements (Model JEM‐2100, 200 kV). X‐ray diffraction (XRD) analysis was performed using a MiniFlex 600 Rigaku diffractometer.

### 2.5 Adsorption Experiments

Adsorption experiments were performed under different conditions with a working volume of 10 mL and at various temperatures (5°C to 28°C), pH (1–9), initial Cr(VI) concentrations (10–100 mg/L), and FeNP‐CC dosage (0.06–0.16 mg). The pH of the working solution was adjusted by adding 0.1N HCl or NaOH solutions. A hot plate and magnetic stirrer were used to control the temperature and stir the solution at 100 rpm during these experiments. Samples (100 µL) were taken at predefined time intervals and analyzed immediately using the 1,5‐diphenylcarbazide method (Method 7196a‐USEPA) [23].

The performance of the FeNP‐CC adsorbent was calculated using the following equation:

(1) $$\mathrm{Removal}\;\mathrm{efficiency}\;\left(\%\right)=\frac{\left(C_{0}-C_{t}\right)}{C_{0}}\times 100$$

where *C*_0_ was the initial Cr(VI) concentration in mg/L and *C*_t_ was the concentration of Cr(VI) remaining in the solution after *t* min.

To assess the kinetics of the Cr(VI) removal process, the pseudo‐first‐order and pseudo‐second‐order models were tested to interpret the experimental data. The rate expression for the pseudo‐first‐order kinetic equation is as follows:

(2) $$\ln\frac{C}{C_{0}}=-K_{1}t$$

where *C* is the concentration of Cr(VI) in solution (mg/L), *C*_0_ is the initial Cr(VI) concentration, *K*_1_ is the pseudo‐first‐order rate constant at adsorption time *t* in minutes. The plot of ln *C*/*C*_0_ versus *t* should give a linear relationship if pseudo‐first‐order rate expression is obeyed [24].

The rate expression for pseudo‐second‐order kinetic equation is as follows:

(3) $$\frac{t}{q_{t}}=\frac{1}{K_{2}{q^{2}}_{e}}+\frac{1}{q_{e}}$$

where *K*_2_ is the pseudo‐second‐order rate constant, *q*_e_ (mg/g) and *q*_t_ (mg/g) are the amounts of Cr(VI) ions removed at equilibrium and at time *t*, respectively:

(4) $$q_{t}=\frac{V\left(C_{0}-C_{t\;}\right)}{m}$$

where *C*_0_ (mg/L), *C*_t_ (mg/L), *V* (mL), and *m* (mg) represent the initial concentration of Cr(VI) ions, the concentration of Cr(VI) after *t* min, the volume of the Cr(VI) solution, and the mass of FeNP‐CC, respectively.

The partition of Cr(VI) ions at equilibrium between solid and liquid phases was modeled using different adsorption isotherms. The Langmuir, Freundlich, and Temkin isotherms were selected for the study [25, 26]. Isotherms were obtained by varying the initial Cr(VI) concentration of 20 to 80 mg/L with 0.16 mg FeNP‐CC at 310 and 318 K for 90 min. Equations for isotherm models are:

Langmuir isotherm:

(5) $$\frac{C_{e}}{q_{e}}=\frac{1}{Q_{0}b}+\frac{C_{e}}{Q_{0}}$$

Freundlich isotherm:

(6) $$\log q_{e}=\log K_{F}+\frac{1}{n}$$

Temkin isotherm:

(7) $$q_{e}=B\ln A_{T\;}+B\ln C_{e}$$

where *C*_e_ = equilibrium solution concentration (mg/L); *q*_e_ = amount of Cr(VI) removed at equilibrium (mg/g); *Q*_0_ = monolayer adsorption capacity (mg/g); *b* = adsorption equilibrium constant (L/mg); *K*_F_ = Freundlich constant for adsorption capacity; *n* = Freundlich constant for adsorption intensity; *B* = Temkin constant; *A*_T_ = equilibrium binding constant.

### 2.6 Statistical Analysis

The data were presented as the mean of sample size n=3 and the standard deviation. The EC50 values for the extract were calculated using linear regression analysis of the concentration–response curves.

## 3 Results and Discussion

### 3.1 Synthesis and Characterization of FeNP‐CC

Upon the addition of extract of *C. cathartica* to 0.01 M FeCl_3_ solution at the volume ratio of 1:2, the color of the iron salt solution changed immediately from yellow to black. This rapid color transition qualitatively indicated the successful formation of FeNP‐CC. The pH of the resulting colloidal FeNP‐CC suspension was 3.4. Under these synthesis conditions, the mass concentration of the synthesized iron nanoparticles was determined to be 0.33 mg/mL. Although the as‐synthesised iron nanoparticles were stable and monodispersed, they exhibited a gradual tendency to aggregate after 2 days; consequently, brief ultrasonication is recommended prior to their application in metal remediation. The FeNP‐CC nanoparticles exhibited a broad UV‐visible absorption maximum at 235 nm (Figure 1). A similar observation was made by Singh et al. for iron nanoparticles, with *λ*_max_ at 235 nm [27]. Notably, the raw extract alone did not display any characteristic absorption peaks in this region.

**FIGURE 1:**
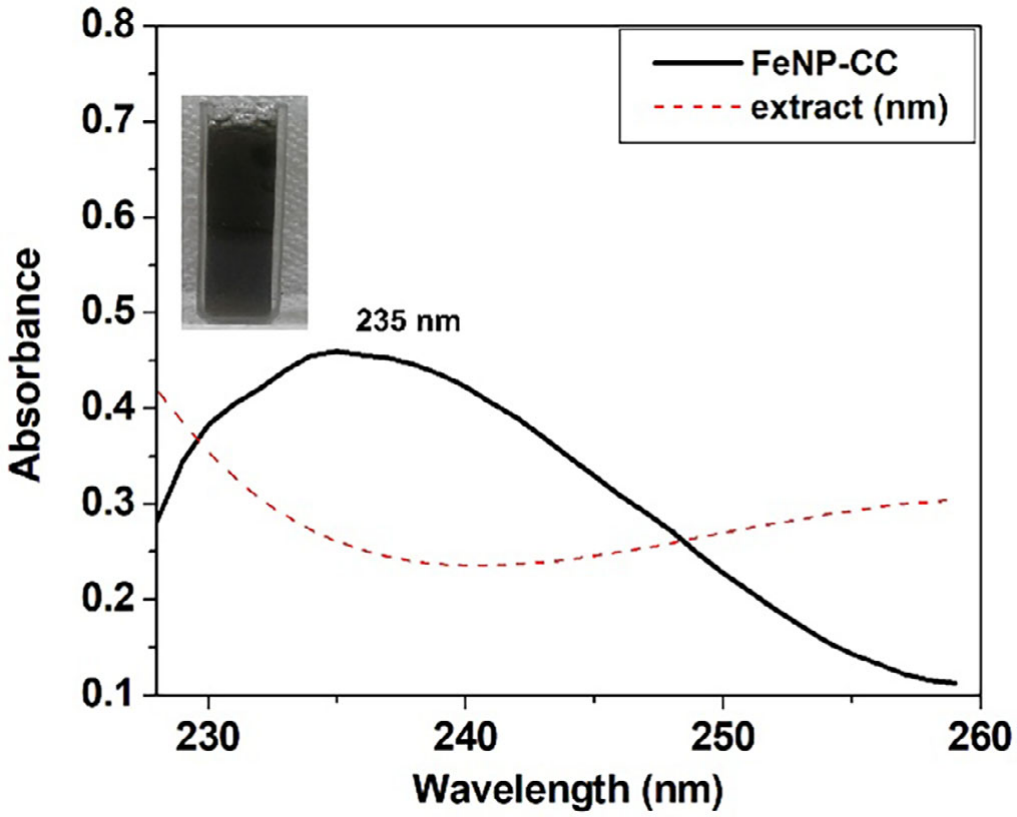
UV‐vis spectrum of FeNP‐CC and extract. Inset photo shows FeNP‐CC.

The zeta potential of the as‐synthesized FeNP‐CC nanoparticles was determined to be −15.84 mV (Figure 2a), indicating a moderately negative surface charge. This negative potential suggests a sufficient level of electrostatic repulsion among the nanoparticles, which helps to maintain moderate colloidal stability in aqueous systems. This net negative surface charge is likely attributed to the surface adsorption of anionic functional groups from polyphenols, proteins, and other capping/stabilizing biomolecules present in the *C. cathartica* seed extract. Such surface charge characteristics are highly advantageous for the electrostatic capture and subsequent adsorption of cationic or oxyanionic heavy metal species such as toxic hexavalent chromium, Cr(VI), via synergistic electrostatic attraction and surface complexation mechanisms.

**FIGURE 2:**
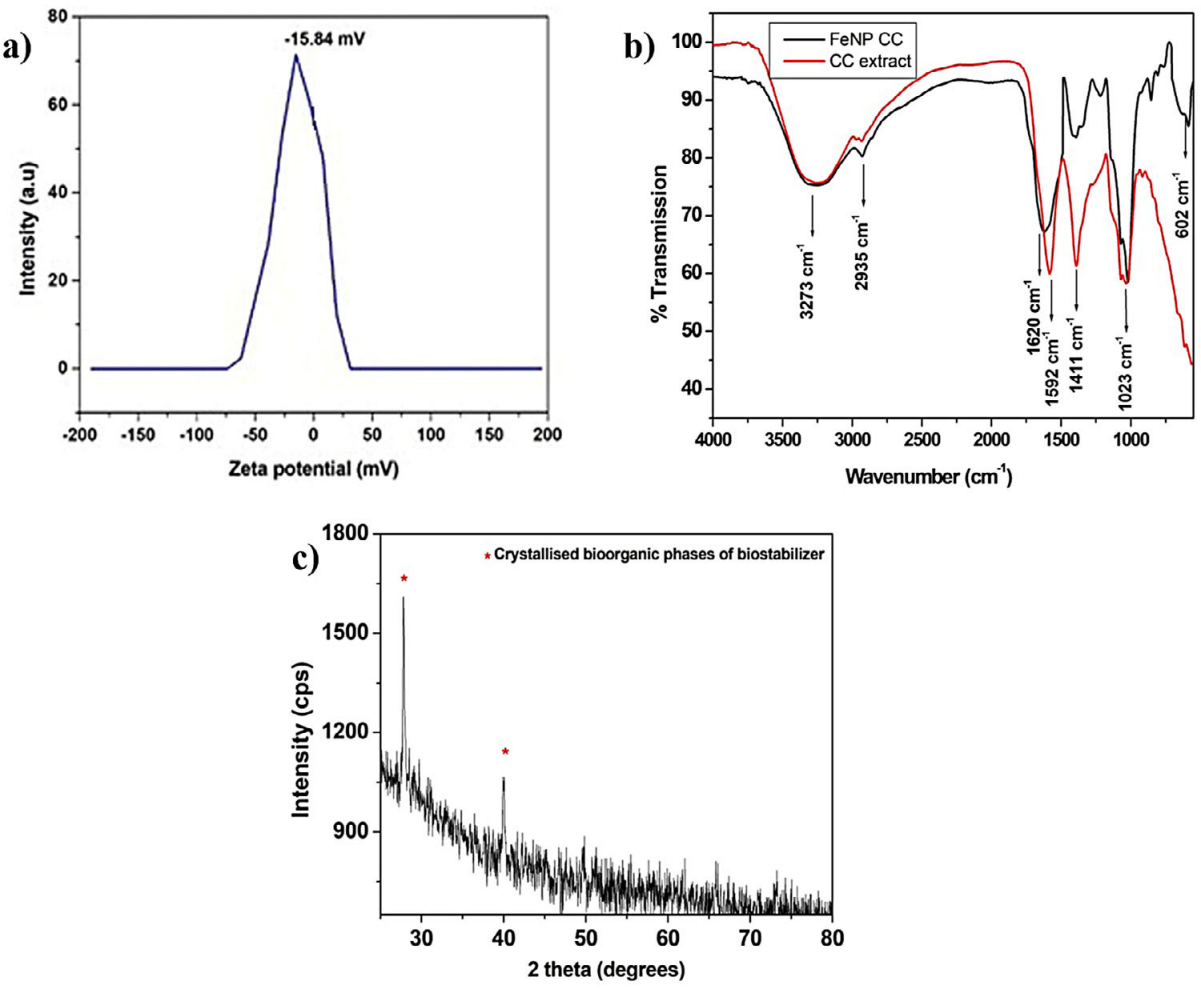
(a) Zeta potential of FeNP‐CC (b) FTIR spectrum of FeNP‐CC and *C. cathartica* extract, and (c) XRD pattern of FeNP‐CC.

Fourier‐transform infrared (FTIR) spectroscopy was performed to identify the specific phytochemical constituents in the *C. cathartica* seed extract responsible for the reduction, capping, and stabilization of the FeNP‐CC nanoparticles (Figure 2b). The bean coats of *Canavalia* species have been reported to contain highly active, bioactive phenolic compounds, such as gallic acid and gallotannins [28]. The FTIR spectra of both the raw extract and the synthesized FeNP‐CC exhibited a broad, intense absorption band centered at 3273 cm^−1^. This prominent band is primarily attributed to the O–H stretching vibrations of free hydroxyl groups from phenolic compounds (such as flavonoids, tannins, and saponins). A weak absorption band observed at 2935 cm^−1^ is assigned to aliphatic C–H asymmetric stretching vibrations. The peak at 1411 cm^−1^ corresponds to the asymmetric C–H bending modes of proteinaceous materials co‐extracted during the process [29]. Furthermore, the intense peak at 1592 cm^−1^ in the raw extract spectrum represents the aromatic ring C═C stretching vibrations [30]. In the FTIR spectrum of the synthesized FeNP‐CC, this aromatic C═C peak shifted to 1620 cm^−1^, strongly indicating a strong chemical coordination between the aromatic/phenolic phytochemicals and the iron nanoparticle surface [31]. The preservation of these primary extract peaks in the nanoparticle spectrum confirms that the key secondary metabolites of *C. cathartica* actively participated as both the reducing agents and the stabilizing, capping matrix during the green synthesis of FeNP‐CC.

XRD data of FeNP‐CC is shown in Figure 2c. The x‐ray diffraction (XRD) analysis of the green‐synthesized FeNP‐CC nanoparticles exhibited two prominent peaks at 2θ ≈ 27° and 40°. The broad peak at ∼27° could be attributed to the semi‐crystalline nature of phytochemical constituents such as polyphenols and gallotannins present in the *C. cathartica* extract. The peak reflected a distorted or nanocrystalline BCC iron (110) plane, indicating a lattice strain or nanoparticle surface modification by capping agents. In BCC zero valent iron nanoparticles, the (110) plane appears at 2θ ≈ 44.7° [32]. However, a peak shift could occur due to phytochemical capping, which appears to be lowered to ∼40°. These features revealed the nanocrystalline nature of synthesized nanoparticles with partial crystallinity originating from the bioorganic components present in the capping/stabilizing agent [33].

Transmission electron microscopy (TEM) provided detailed insights into the morphology, size, and structural characteristics of the synthesized iron oxide nanoparticles. As observed in the low‐magnification TEM images (Figure 3a–b), the nanoparticles were uniformly dispersed, exhibiting a spherical morphology with minimal aggregation, indicating effective stabilization during the synthesis process. Upon closer examination at higher magnification (20 nm scale, Figure 3b), the nanoparticles appeared to be symmetrically arranged in a circular manner on the surface of larger ball‐like structures, which were supposed to be biomolecular assemblies responsible for stabilizing the iron nanoparticles. The average particle size, calculated from the TEM images, was 6.02 ± 1.32 nm (Figure 3c).

**FIGURE 3:**
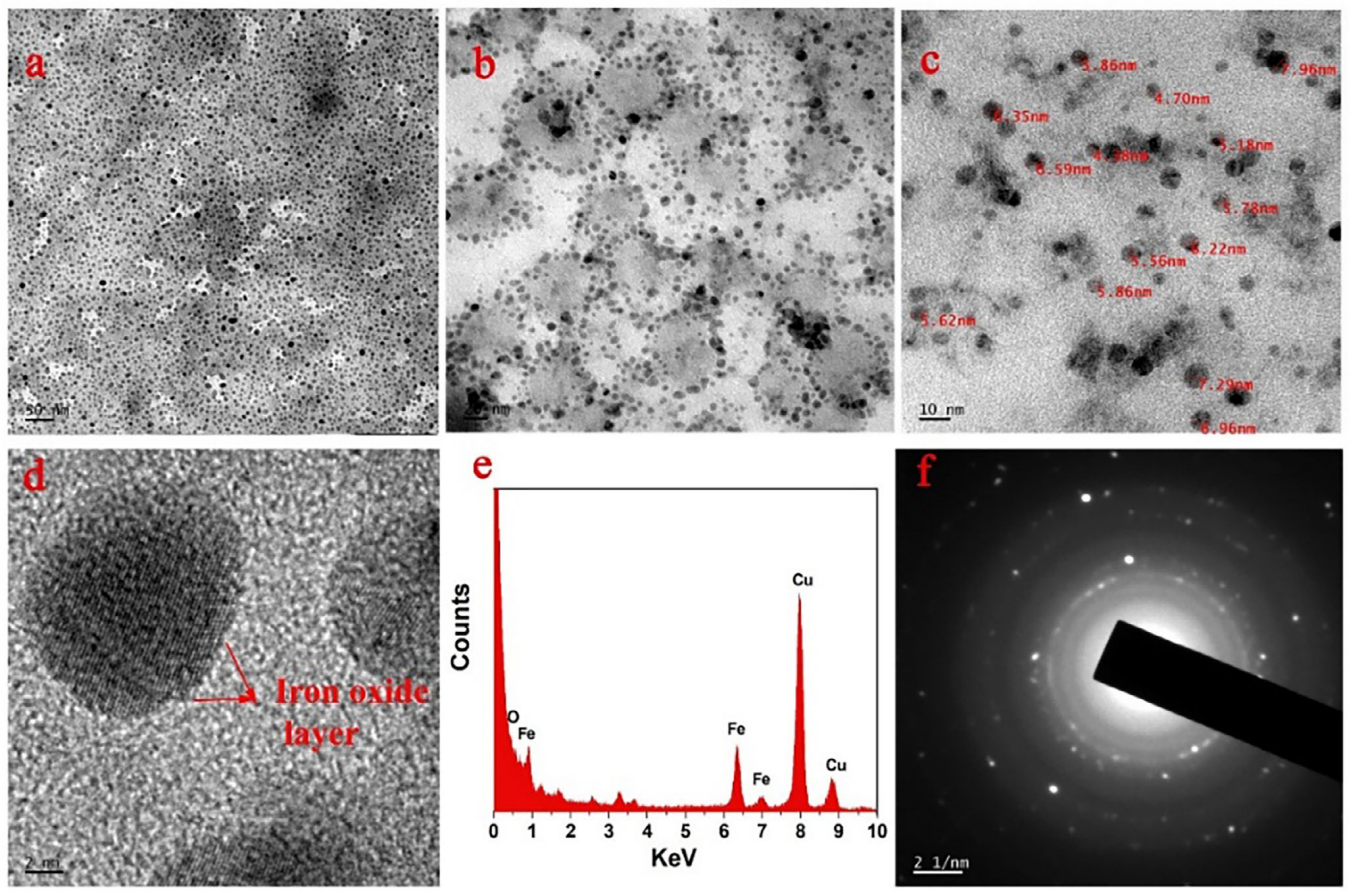
(a–d) shows the TEM images, (e) EDX pattern, and (f) SAED pattern of FeNP‐CC.

High‐resolution TEM (HRTEM) imaging of an individual FeNP‐CC (Figure 3d) revealed distinct lattice fringes, confirming a crystalline internal structure. A core–shell contrast was evident, wherein a lighter shell surrounded a darker core—attributed to an iron oxide layer enveloping a metallic or mixed‐valence iron core. This structural feature suggests surface‐associated oxidation and passivation of the zero‐valent iron nanoparticles.

Energy‐dispersive x‐ray spectroscopy (EDX) analysis (Figure 3e) showed prominent peaks for iron (Fe) and oxygen (O), confirming the formation of iron oxide. The intense Fe signal supports the presence of an iron‐rich core, while the O peak indicates surface‐associated oxidation, in excellent agreement with the core–shell architecture observed via HRTEM. Based on this evidence, it was inferred that the FeNP‐CC predominantly consisted of a crystalline iron core surrounded by an amorphous or partially oxidized iron oxide shell. This structural interpretation is highly consistent with the findings of Krajewski et al., who documented the formation of iron nanoparticles featuring an intermediate amorphous iron layer between the crystalline core and the outer oxidized surfaces [34].

The crystallinity of the nanoparticles was further confirmed by the selected area electron diffraction (SAED) pattern (Figure 3f), which exhibited sharp, well‐defined concentric rings; characteristic of polycrystalline structures. The estimated weight and atomic percentages of iron (Fe) and oxygen (O) present in the nanoparticles were calculated by normalizing the concentrations of these two primary elements, thereby excluding background signals such as the copper (Cu) peak originating from the TEM support grid (Table 1).

**TABLE 1:** Weight percentages and atomic percentages of iron and oxygen in FeNP‐CC, as determined by EDX measurements.

Element	Weight %	Atomic %
O K	7.24	21.42
Fe K	92.76	78.58
Total	100.00	

*Note*: The copper (Cu) peak arising from the sample support grid was excluded from the table to ensure accurate estimation of the total elemental weight percentage of the sample.

Together, the HRTEM, EDX, SAED, and XRD analyses confirmed that the synthesized FeNP‐CC exhibited a core–shell structure comprising pure Fe–O phases, with a crystalline core and an amorphous or oxidized shell.

### 3.2 Phytochemical Analysis of *C. cathartica* Extract

Preliminary screening of the aqueous seed extract of *C. cathartica* confirmed the presence of flavonoids, proteins, saponins, and tannins, whereas alkaloids and cardiac glycosides were absent. The total phenolic content (TPC) was quantified at approximately 125 mg GAE/g dry weight (Figure S1). To assess the reductive and electron‐donating capacity of these constituents, ferric reducing antioxidant power (FRAP) and total antioxidant capacity (TAC) assays were conducted.

The FRAP assay demonstrated a concentration‐dependent reducing power of 3.56 ± 0.28 µg/mL, compared to that of standard vitamin E (0.62 ± 0.06 µg/mL) (Figure S2). Based on the linear calibration curve of ferrous sulfate (*y* = 0.00314*x* + 0.00153, *R* ^2^ = 0.9947), the extract exhibited a high reducing power of 1256.57 ± 8.59 mg/g TE (Figure S3). Correspondingly, phosphomolybdenum‐based TAC assays—quantified using a Trolox standard curve (*y* = 0.0152*x*, *R* ^2^ = 0.98463)—revealed an exceptional total reducing capacity, where 1 g of *C. cathartica* seed extract (Figure S4) was equivalent to 1.03 g of Trolox. These results highlight the strong electron‐donating capacity of the extract, facilitating the rapid reduction of Fe^3+^ ions to Fe°.

Liquid Chromatography–Tandem Mass Spectrometry (LC‐MS/MS) analysis of the aqueous seed extract of *Canavalia cathartica* was conducted in both positive and negative electrospray ionization (ESI) modes (Figure 4). The total ion chromatogram (TIC) in positive mode exhibited a dominant peak eluting between 1.0 and 1.5 min, suggesting the presence of a highly polar compound.

**FIGURE 4:**
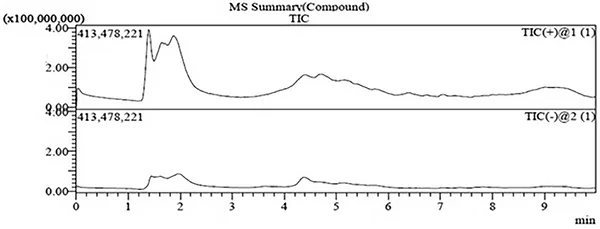
Mass chromatogram of *C. cathartica* extract.

To identify the specific biomolecules driving this reducing and capping efficiency, liquid chromatography–tandem mass spectrometry (LC‐MS/MS) was performed in both positive and negative electrospray ionization (ESI) modes (Figure 4). The positive‐mode total ion chromatogram (TIC) exhibited a dominant peak eluting early between 1.0 and 1.5 min, indicating a highly polar major constituent.

This major compound displayed a prominent protonated molecular ion [M + H]^+^ at m/z 177, corresponding to the theoretical mass of L‐canavanine (C_5_H_13_N_3_O_3_, MW 175.17 g/mol) (Figures S5 and S6). Product ion scanning of m/z 177generated diagnostic fragment ions at m/z160 (due to the loss of NH_3_) and m/z 102, (a well‐characterized diagnostic ion for L‐canavanine). Importantly, the absence of the characteristic arginine fragment at m/z 129 further confirmed this structural assignment. Collectively, the chromatographic retention, ESI behavior, and MS/MS fragmentation profile (Figure 5) conclusively confirm the presence of L‐canavanine as the principal active nitrogenous capping agent in the extract.

**FIGURE 5:**
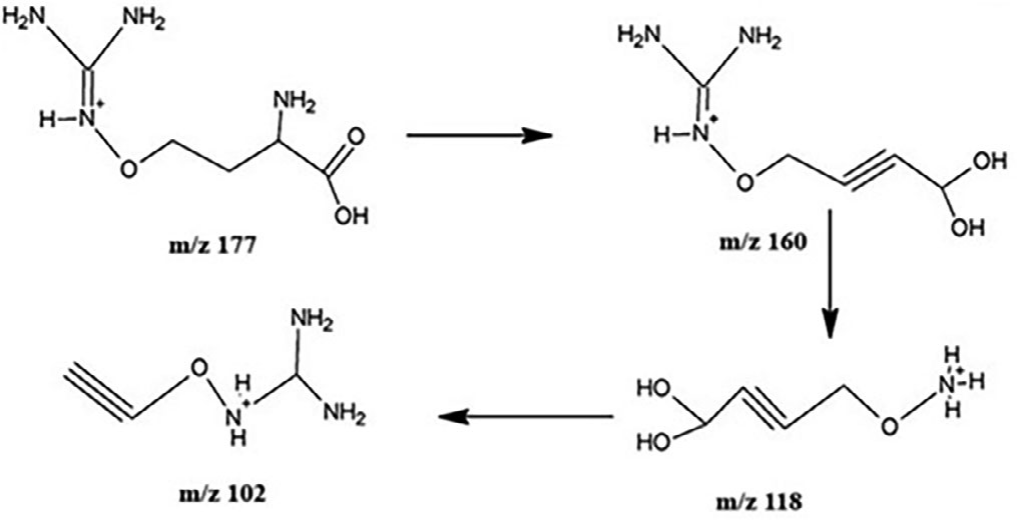
Proposed fragmentation of canavanine.

Therefore, the qualitative and quantitative phytochemical screening of the *C. cathartica* extract, complemented by FTIR and LC‐MS/MS analyses, confirmed the presence of various bioactive constituents, including flavonoids, proteins, saponins, tannins, and notably, L‐canavanine—a non‐proteinogenic amino acid structurally analogous to arginine. These phytochemicals contain phenolic, amino, carboxyl, and guanidino functional groups, which are known to exhibit strong affinity toward metal ion surfaces. In the context of nanoparticle synthesis, such groups can function as both reducing agents, by donating electrons to metal precursors, and capping agents, by binding to the nanoparticle surface and preventing aggregation. Specifically, the reactive guanidino, amino, and carboxyl groups of L‐canavanine are likely to facilitate reduction of iron ions and simultaneously provide electrostatic and hydrogen‐bonding interactions, thereby contributing significantly to the stabilization of the iron nanoparticles [35].

This exceptional quantitative antioxidant profile is directly responsible for driving the thermodynamic reduction and subsequent stabilization of the nanoparticles. The reduction of precursor Fe^3+^ ions to Fe° requires an electron‐donating matrix capable of overcoming demanding standard reduction potentials. The abundance of hydroxylated phenolic compounds in our extract (125 mg GAE/g) provides a highly concentrated pool of reactive O–H groups that readily undergo oxidation, donating electrons to the transition Fe^3+^ precursor into nucleating metallic nanoparticles. Following core nucleation, the oxidized phenolic structures, alongside proteins and L‐canavanine, act as active capping ligands. These molecules coordinate onto the newly formed metallic surfaces—as evidenced by the shifting of the aromatic C═C FTIR band to 1620 cm^−1^—and impart a moderately negative surface potential of −15.84 mV. This surface functionalization establishes a crucial electrostatic barrier that maintains colloidal stability and prevents rapid nanoparticle aggregation.

### 3.3 Effect of Physicochemical Parameters on Cr(VI) Removal

#### 3.3.1 Comparative Performance of FeCl_3_, Seed Extract, and FeNP‐CC

To evaluate the efficacy of the synthesized nanobiosorbent, the Cr(VI) removal efficiencies of pure ferric chloride (FeCl_3_), the raw *Canavalia cathartica* seed extract, and the biogenic FeNP‐CC were compared using a 10 mg/L Cr(VI) solution over a 30‐min reaction period. Under identical experimental conditions, pure FeCl_3_ exhibited negligible removal, accounting for only about 10% of Cr(VI). Thermodynamically, Fe^3^^+^ cannot act as a direct reductant for hexavalent chromium; however, under the acidic conditions of the reaction, a minor fraction of Fe^3^^+^ was likely reduced to Fe^2^^+^, which subsequently mediated the partial reduction of Cr(VI) to Cr(III) [36]. In comparison, an equivalent amount of extract achieved approximately 23% Cr(VI) removal within 30 min. This removal was attributed to the extract's inherent adsorption capability or its reducing nature.

FeNP‐CC demonstrated significantly enhanced Cr(VI) removal, reaching nearly 98% within the same time frame. This substantial improvement was attributed to the synergistic action between the iron nanoparticles and the phytochemicals present in the extract, which facilitated both adsorption and redox processes.

#### 3.3.2 Effect of FeNP‐CC Dosage

The impact of FeNP‐CC dosage on Cr(VI) removal was systematically investigated by varying the adsorbent concentration from 0.06 mg to 0.16 mg (Figure 6(a)). Within a contact time of 35 min, the Cr(VI) removal efficiency increased dramatically from 41.4% to 98.43% as the dosage was raised from 0.06 mg to 0.16 mg. Complete Cr(VI) removal (100%) was achieved within 50 min at a dosage of 0.14 mg or higher. This positive correlation between adsorbent dosage and removal efficiency is directly ascribed to the greater availability of active surface sites and the increased total specific surface area offered by the larger mass concentration of the nanoparticles in the aqueous system.

**FIGURE 6:**
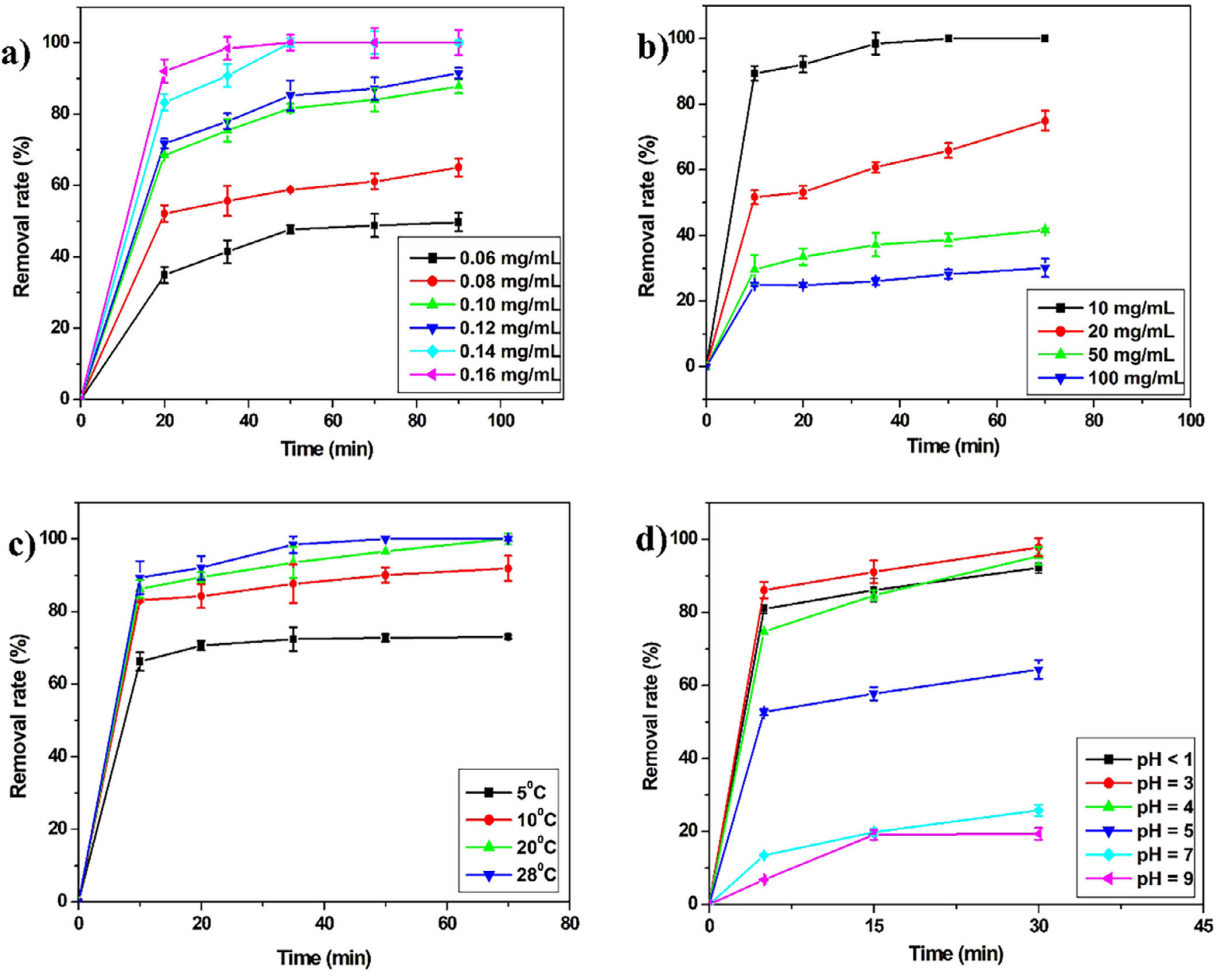
Effect of various factors on Cr(VI) removal: (a) dosage of FeNP‐CC, (b) initial Cr(VI) concentration, (c) reaction temperature, and (d) pH value. Error bars represent the standard deviation of triplicate measurements.

#### 3.3.3 Effect of Initial Cr(VI) Concentration

The influence of the initial Cr(VI) concentration was examined across a range of 10 to 100 mg/L (Figure 6b). It was observed that the percentage removal of Cr(VI) was inversely proportional to its initial concentration. While complete (100%) removal was successfully achieved for a 10 mg/L Cr(VI) solution within 50 min, the removal efficiency declined by approximately 37% when the initial concentration was increased to 20 mg/L. A further increase to 100 mg/L resulted in a substantial 68% reduction in overall removal efficiency. This downward trend is attributed to the saturation of a fixed number of active adsorption/reduction sites on the FeNP‐CC surface. At higher initial concentrations, the ratio of available surface sites to the total Cr(VI) ions in solution decreases, leaving a significant portion of the oxyanions unreacted in the aqueous phase.

#### 3.3.4 Effect of Reaction Temperature

The influence of reaction temperature on Cr(VI) removal was evaluated between 5°C and 28°C (Figure 6c). The results indicate that elevated temperatures significantly promote the remediation process, showing an approximate 25% increase in removal efficiency within 90 min as the temperature was raised from 5°C to 28°C. According to classical collision theory, higher temperatures increase the kinetic energy of both the FeNP‐CC adsorbent and the Cr(VI) oxyanions. This kinetic activation leads to a greater collision frequency and overcomes the activation energy barrier of this surface‐mediated redox reaction [37]. Furthermore, the elevated temperature accelerates the rate of interfacial electron transfer from the zero‐valent/mixed‐valence iron core of the FeNP‐CC to the adsorbed Cr(VI) species, thereby facilitating rapid chemical reduction.

#### 3.3.5 Effect of Initial Solution pH

The initial pH of the solution is a critical parameter governing both the surface charge characteristics of the adsorbent and the ionic speciation of chromium in water. The influence of pH was evaluated across a range of 1.0 to 9.0 (Figure 6d). The results clearly demonstrate that Cr(VI) removal is highly favored under acidic conditions and decreases drastically in alkaline media. Chromium speciation is highly sensitive to pH in aqueous media. Cr(VI) was more effectively removed under acidic conditions compared to alkaline media. Cr(VI) existed primarily as H_2_CrO_4_ (at pH < 1.0), HCrO_4_^−^ (between pH 1.0 and 6.0), and CrO_4_^2^^−^ (at pH > 6.0) [38]. The removal efficiency at various pH levels was found to depend on both the electrostatic interactions between Cr(VI) species and the FeNP‐CC surface, as well as the type of Cr(III) precipitate formed after reduction.

At acidic pH values (pH 1.0 to 6.0), the amine, carboxyl, and guanidine functional groups of the biomolecules (such as L‐canavanine and co‐extracted proteins) capping the FeNP‐CC surface undergo intensive protonation, forming positively charged species such as NH_3_^+^, COOH_2_^+^, and protonated guanidinium groups. This positive surface charge establishes a strong electrostatic attraction toward the dominant anionic HCrO_4_^−^ species, drawing them to the nanoparticle surface for subsequent adsorption and reduction. At pH < 1.0, the dominant species shifts to neutral H_2_CrO_4_, which lacks a net charge, resulting in a slight decrease in electrostatic attraction compared to pH 3.0 (Figure 6d). Within 30 min, over 90% Cr(VI) removal was achieved in all experiments conducted at pH below 4.0. Conversely, at pH values above 7.0, the removal efficiency dropped below 20%. This sharp decline is attributed to the deprotonation of the capping biomolecules, which imparts a net negative charge to the FeNP‐CC surface, resulting in strong electrostatic repulsion against the anionic CrO_4_^2^^−^ ions, while also increasing competitive adsorption from hydroxyl OH^−^ ions.

Furthermore, the solution pH directly affects the solubility and nature of the reduced trivalent chromium [Cr(III)] species formed on the nanoparticle surface. Cr(III) typically precipitates as chromium hydroxide Cr(OH)_3_, iron‐chromium hydroxide Fe(III)–Cr(III) hydroxide, or mixed oxyhydroxides [39]. Under highly acidic conditions, the reduced Cr(III) forms more soluble or dispersed species, leaving the FeNP‐CC active sites exposed for continuous reactions. However, at higher pH values, insoluble mixed‐phase Fe(III)–Cr(III) oxyhydroxides rapidly precipitate and deposit directly onto the FeNP‐CC surface. This mineral precipitation forms a dense, passive layer that blocks the active sites and hinders further interfacial electron transfer, thereby suppressing both the adsorption and reduction of remaining Cr(VI) ions. Consequently, an acidic pH (optimum at pH 4.0) is essential for maintaining both high electrostatic attraction and preventing surface passivation.

### 3.4 Kinetics Study of the Reaction

The adsorption kinetics of Cr(VI) onto the green‐synthesized FeNP‐CC were investigated to elucidate the underlying adsorption mechanisms and rate‐limiting steps. The experimental kinetic data were analyzed using both pseudo‐first‐order (Equation 2) and pseudo‐second‐order (Equation 3) models. As illustrated in Figure 7a,b, a significant difference in model fitting was observed, with the pseudo‐second‐order model showing a substantially higher correlation with the experimental data across all studied adsorbent dosages.

**FIGURE 7:**
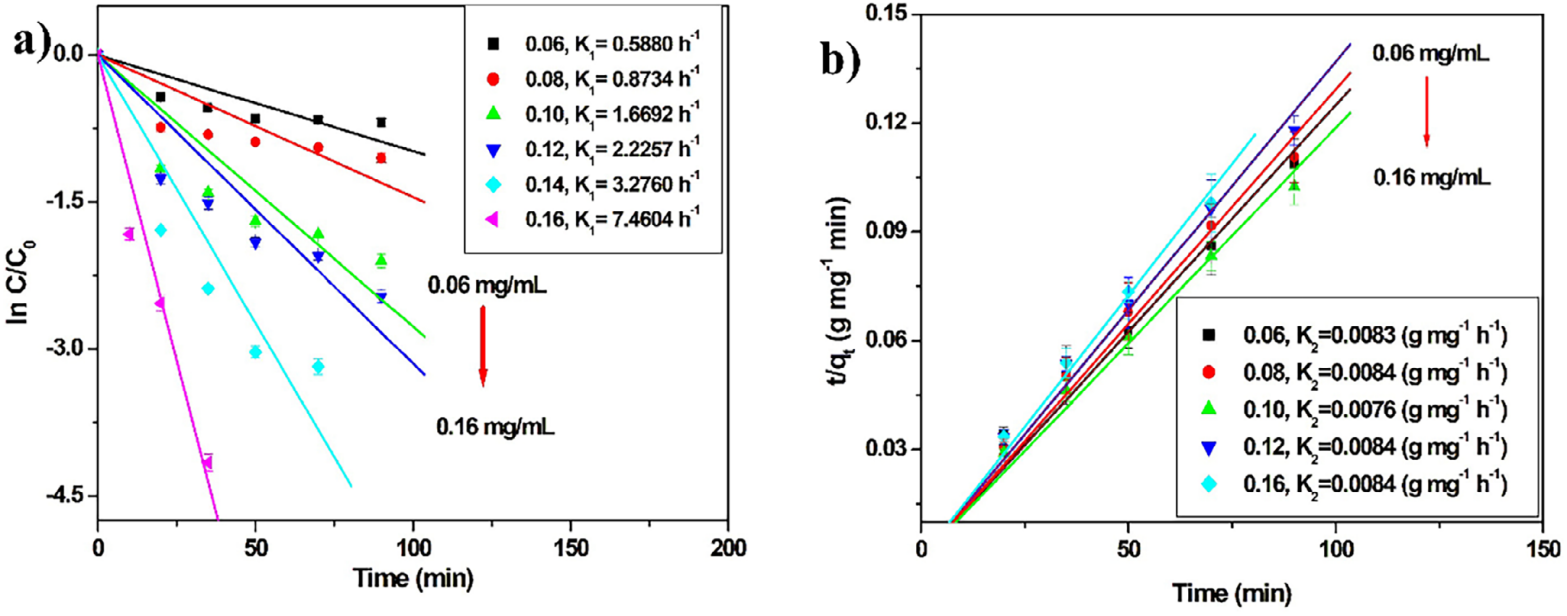
(a) Pseudo‐first‐order kinetic model and (b) pseudo‐second‐order kinetic model for Cr(VI) removal. Error bars represent the standard deviation of triplicate measurements.

The pseudo‐first‐order model, which assumes a physical adsorption mechanism governed by diffusion‐limited rate control, yielded poor correlation coefficients (*R* ^2^ values ranging from 0.8444 to 0.9360). The non‐linear deviation observed in the plots of ln(C/C_0_) versus time indicates that the pseudo‐first‐order model is inadequate for representing the Cr(VI) removal kinetics in this system, particularly at higher FeNP‐CC dosages and extended reaction intervals.

In contrast, the pseudo‐second‐order kinetic model demonstrated exceptional linearity, with correlation coefficients (*R* ^2^) exceeding 0.99 under all investigated experimental conditions. This excellent mathematical fit strongly implies that the adsorption of Cr(VI) onto the FeNP‐CC surface is predominantly governed by a chemisorption mechanism. This process likely involves chemical valence forces through electron sharing, transfer, or coordinate bonding between the chromium species and the various surface‐bound functional groups. The biogenic FeNP‐CC, prepared using *C. cathartica* seed extract, is richly capped with active biomolecules containing phenolic, amino, carboxylic, and guanidinium moieties. These functional groups act as highly reactive coordination sites, facilitating strong ligand‐mediated complexation and surface redox reactions with Cr(VI) species.

Furthermore, the calculated pseudo‐second‐order rate constants, *K*_2_ (around 0.008 g mg^−1^ h^−1^) remained highly consistent across the studied adsorbent dosages, confirming the reliability and robustness of the second‐order kinetic model. The rapid initial adsorption rates observed during the first 20–30 min are attributed to the abundant availability of vacant active sites on the freshly dispersed FeNP‐CC surface. As the reaction progressed toward equilibrium, these active sites became progressively saturated, leading to a plateau in the Cr(VI) removal curves beyond 60 min. Consequently, a contact time of 90 min was selected as the optimal equilibrium duration for the subsequent adsorption isotherm and thermodynamic investigations.

### 3.5 Adsorption Isotherms

To evaluate the equilibrium partition of Cr(VI) ions between the solid and liquid phases, the experimental data obtained at an equilibrium contact time of 90 min were fitted to the Langmuir (Equation 5), Freundlich (Equation 6), and Temkin (Equation 7) isotherm models at 310 and 318 K (Figure 8). Among these, the Freundlich isotherm model yielded the most accurate representation of the equilibrium data, with high correlation coefficients *R* ^2^ of 0.9826 and 0.9920 at 310 and 318 K, respectively. This superior fit indicates that Cr(VI) removal is governed by a multilayer, heterogeneous adsorption mechanism on the non‐uniform surface of the nanoparticles [40]. This surface heterogeneity is directly attributed to the structurally diverse capping biomolecules—such as L‐canavanine, flavonoids, and tannins—which impart a variety of functional groups (including hydroxyl, amino, and carboxylate moieties) to the FeNP‐CC surface, providing a range of binding sites with varying adsorption energies [41].

**FIGURE 8:**
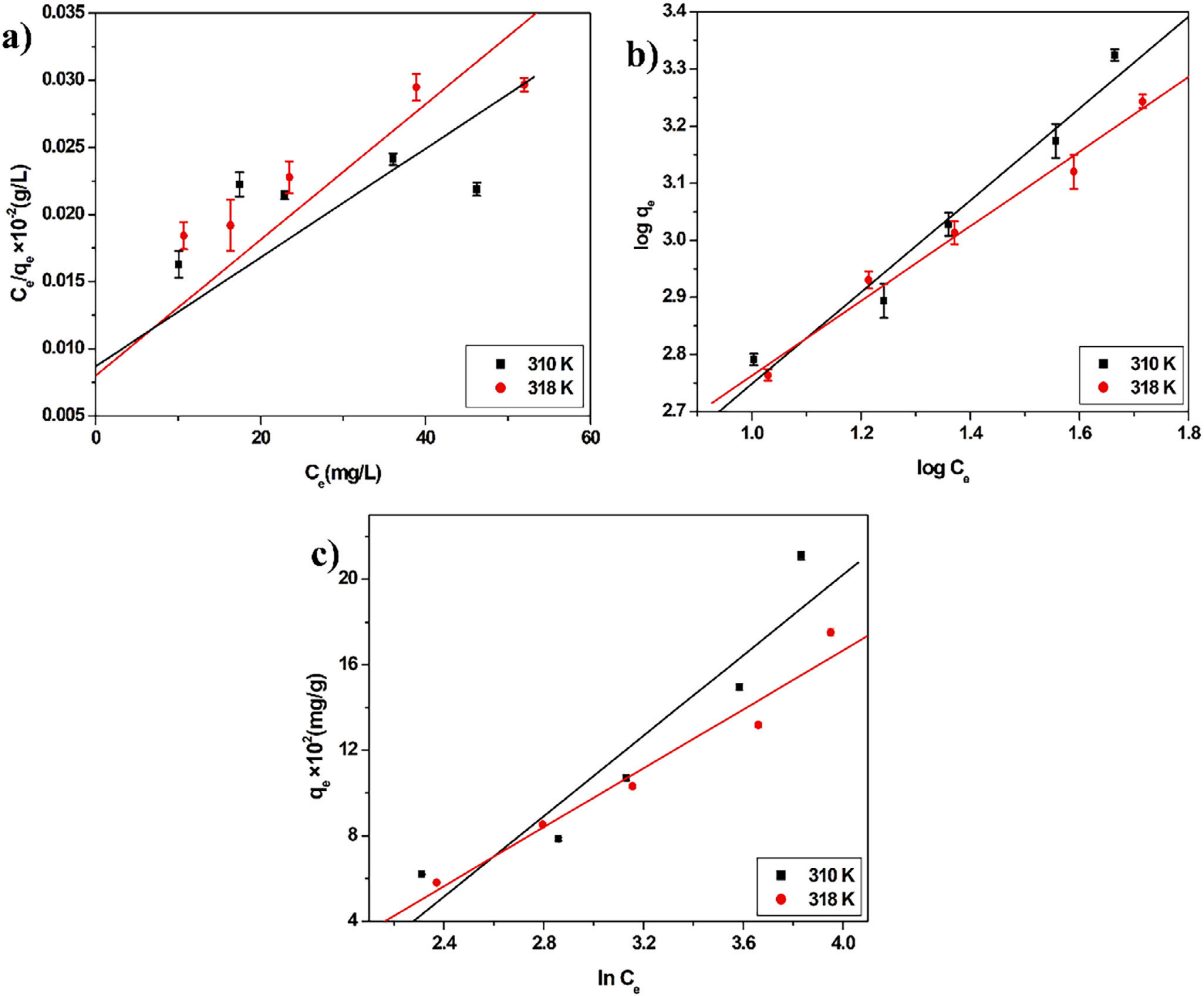
Linearized (a) Langmuir, (b) Freundlich, and (c) Temkin isotherms for Cr(VI) removal by FeNP‐CC at 310 K and 318 K. Error bars represent the standard deviation of triplicate measurements.

The Freundlich capacity constant (K_F_), which reflects the relative adsorption capacity, increased 88.30 mg/g at 310 K to 128.50 mg/g at 318 K. This increase confirms that the adsorption capacity is enhanced at elevated temperatures, pointing to a thermodynamically favorable, endothermic process [42]. Moreover, the adsorption intensity parameter (*n*), determined from the reciprocal slope of the Freundlich plot (log *q*_e_ against log *C*_e_), was found to be 1.2445 at 310 K and 1.5304 at 318 K. Since both values are greater than unity (*n* > 1), they indicate highly favorable adsorption under the investigated temperature range.

Compared to various other adsorbents reported in recent literature (Table 2), the green‐synthesized FeNP‐CC displays remarkably superior K_F_ values and adsorption affinities. This exceptional performance firmly establishes FeNP‐CC as a highly effective, thermally stable, and chemically interactive material for the remediation of toxic hexavalent chromium from contaminated aqueous systems.

**TABLE 2:** Comparison of Cr(VI) adsorption capacity of different adsorbent types reported in literature.

Adsorbent	Temperature (K)	K_F_ (mg/g)	Reference
Fermented sweet sorghum residues	303	0.83–4.76	[43]
*Rhaponticum repens* (L.) Hidalgo‐based Fe nanoparticles	298	167.6	[44]
Magnetic Fe_3_O_4_ sludge	303	135.4	[45]
Fe_2_O_3_‐chitosan‐cherry kernel shell	298	47.57	[46]
Iron‐doped chlorapatite‐modified biochar	298	12.65	[47]
SDS–nZVI	313	20.51	[48]
	343	92.74	
Nano‐Fe_3_O_4_/waste banana peel/alginate hydrogel biobeads	298	2.886	[49]
Graphene oxide/chitosan/ferrite nano composites	298	48.26	[50]
Fe_2_O_3_ nanocomposites	298	24.06	[51]
GZnO/PPAC	298	6.396	[52]
Graphene oxide from *Strychnos potatorum* seeds	298	9.09	[53]
Mesoporous hydrochar from Acacia falcata leaves	303	2.182	[54]
*S. cerevisiae*/alginate composite	298	72.1	[55]
FeNP‐CC	310	88.30	Present work
	318	128.50	

### 3.6 Proposed Mechanism of Cr(VI) Removal by FeNP‐CC

FeNP‐CC mediates the removal of toxic hexavalent chromium, Cr(VI), through a synergistic dual adsorption–reduction mechanism. This process is driven by the highly reactive core–shell structure of the nanoparticles, combining the electron‐donating capacity of the zero‐valent/mixed‐valence iron core with the diverse chemical functional groups provided by the *Canavalia cathartica* seed extract. The overall remediation process proceeds through four interconnected stages: electrostatic attraction, ligand‐mediated surface complexation, surface‐mediated redox reduction, and subsequent surface precipitation or co‐precipitation.

#### 3.6.1 Protonation and Electrostatic Attraction

Under highly acidic conditions (pH 2.0–4.0), the surface‐bound biomolecular capping matrix of FeNP‐CC undergoes extensive protonation. The hydroxyl (–OH), amino (–NH_2_), and carboxyl (–COOH) groups from the co‐existing L‐canavanine, flavonoids, and proteins are converted into their respective positively charged forms (–OH_2_^+^, –NH_3_^+^, and –COOH_2_^+^). This dense, positive surface charge creates a strong electrostatic gradient that attracts anionic Cr(VI) species, which predominantly exist as HCrO_4_^−^ and Cr_2_O_7_^2^^−^ in this pH range [56]. This electrostatic capture serves as the initial rate‐accelerating step, rapidly accumulating the toxic oxyanions at the nanoparticle‐water interface.

#### 3.6.2 Ligand‐Mediated Surface Complexation

Beyond pure electrostatic interactions, the functional groups from the *C. cathartica* extract—particularly the reactive guanidino, amino, and carboxylate moieties of L‐canavanine, alongside the phenolic groups of flavonoids and tannins—act as highly active coordinating ligands. These electron‐rich groups form stable inner‐sphere complexes with the adsorbed chromium ions through ligand‐to‐metal charge transfer and orbital overlap on the nanoparticle surface [57]. This strong chemical bonding establishes a strong surface complexation network, preventing the desorption of the captured chromium and aligning with the pseudo‐second‐order kinetic model, which highlights chemisorption as the rate‐limiting step.

#### 3.6.3 Surface‐Mediated Redox Reduction

Simultaneously, the zero‐valent or mixed‐valence iron core of the FeNP‐CC functions as an efficient, highly active electron donor [58]. Electrons are transferred from the iron core through the thin, bio‐capped oxide shell to the adsorbed Cr(VI) species. This thermodynamically favorable surface redox reaction reduces toxic, highly mobile Cr(VI) to the significantly less toxic and immobile trivalent state, Cr(III).

This electron‐transfer pathway is further assisted by the antioxidant secondary metabolites (polyphenols and flavonoids) present in the capping layer. These biomolecules not only facilitate the reduction of iron ions during synthesis but also helps to stabilize the intermediate chromium oxidation states through secondary coordination or hydrogen bonding.

#### 3.6.4 Surface Precipitation and Heterogeneous Multilayering

Following the redox transformation, the newly generated Cr(III) species precipitate on the nanoparticle surface [59]. Depending on the local pH and ionic microenvironment at the interface, Cr(III) precipitates as insoluble chromium hydroxide [Cr(OH)_3_] or is co‐precipitated with oxidized iron species [Fe(III)] to form highly stable mixed‐phase Fe(III)–Cr(III) hydroxides or oxyhydroxides [60].

The structural and chemical heterogeneity of the FeNP‐CC surface—resulting from the multi‐functional phytochemical capping shell—provides a diverse spectrum of adsorption sites with varying binding affinities. This chemical diversity facilitates a multilayer, site‐specific adsorption process across the heterogeneous surface, as mathematically validated by the excellent fit of the equilibrium data to the Freundlich isotherm model.

### 3.7 Application of FeNP‐CC for the Remediation of Cr(VI) from Real Laboratory Wastewater

To evaluate its practical applicability, FeNP‐CC was deployed to treat complex laboratory wastewater collected from the School of Environmental Sciences, Mahatma Gandhi University, Kottayam, Kerala, India, which was diluted to a Cr(VI) concentration of 30 mg/L (Figure 9). This wastewater, primarily generated during routine chemical oxygen demand (COD) tests and argentometric titrations, contains significant quantities of discharged chromium species. Compared to a synthetic, spiked Cr(VI) standard solution, a decrease of over 20% in the Cr(VI) removal rate was observed under identical operating conditions. This reduction in remediation efficiency is directly attributed to matrix interferences from co‐existing cations, anions, and dissolved natural organic matter.

**FIGURE 9:**
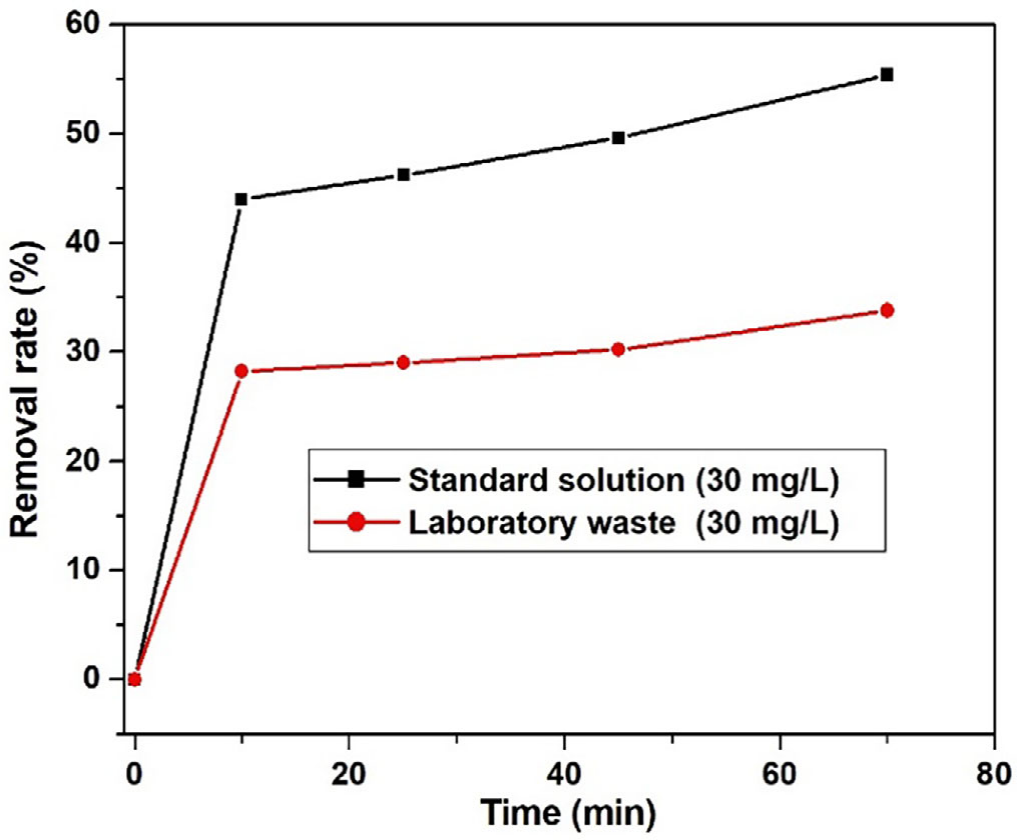
Comparison of removal capacity between laboratory sample and spiked sample.

Specifically, competing anions—such as phosphate, nitrate, carbonate, and sulfate—directly compete with anionic Cr(VI) species for the positively charged, protonated active sites on the FeNP‐CC surface [61]. Furthermore, ubiquitous humic acid (HA) adsorbs onto the nanoparticles, blocking active surface sites and inducing steric hindrance that limits adsorbate diffusion. The high ionic strength of the real wastewater matrix also compresses the electrical double layer (EDL) of the FeNP‐CC, reducing electrostatic repulsion and promoting nanoparticle aggregation, which subsequently decreases the net active surface area [62]. Additionally, co‐existing heavy metal cations and digestion reagents act as competing electron acceptors for the reactive zero‐valent iron core, accelerating surface passivation via the deposition of mixed‐phase oxyhydroxides. Despite these substantial matrix interferences, FeNP‐CC successfully remediated the real wastewater, confirming its viability for practical, real‐world environmental treatment.

## 4 Conclusions

In this study, biogenic iron nanoparticles (FeNP‐CC) were successfully synthesized via an eco‐friendly green route using the aqueous seed extract of the mangrove associate *Canavalia cathartica*. The plant's unique, stress‐adapted phytochemical profile—specifically rich in L‐canavanine and hydroxylated phenolic compounds—served a dual role as both a thermodynamic reducing agent and an interfacial capping stabilizer. Comprehensive characterization (UV‐Vis, FTIR, XRD, HR‐TEM, and EDX) confirmed that the as‐synthesized FeNP‐CC possesses a nanocrystalline core–shell architecture (average particle size 6.02 ± 1.32 nm) comprising a crystalline metallic iron core enveloped by a partially oxidized, bio‐capped amorphous shell with moderate colloidal stability (zeta potential ‐15.84 mV). Under optimized acidic conditions (pH 4.0), the biogenic nanoparticles demonstrated exceptional efficiency, achieving complete (100%) removal of a 10 mg/L Cr(VI) solution within 50 min.

Kinetic and equilibrium modeling revealed that the Cr(VI) remediation process is governed by a pseudo‐second‐order kinetic model, confirming chemisorption involving chemical valence forces and ligand‐mediated surface complexation as the rate‐limiting step. The equilibrium partition data fitted exceptionally well to the Freundlich isotherm model, indicating a multilayer, heterogeneous adsorption mechanism across the non‐uniform surface of the nanoparticles. The Freundlich capacity constant significantly increased from 88.3 mg/g at 310 K to 128.50 mg/g at 318 K, confirming that the endothermic remediation process is thermodynamically favored at elevated temperatures.

From a practical and economic standpoint, utilizing the locally abundant, neglected mangrove associate *C. cathartica* offers an exceptionally sustainable, cost‐effective, and low‐energy synthesis platform ideal for low‐resource settings. Although real laboratory wastewater evaluations revealed an approximate 20% reduction in removal kinetics due to matrix interferences—including competitive anionic adsorption (phosphate, nitrate, carbonate, and sulfate), humic acid shielding, and ionic strength‐driven double‐layer compression—the FeNP‐CC nanoparticles successfully treated the wastewater, confirming their viability for real‐world environmental applications. To overcome the challenges of colloidal aggregation and passive surface fouling, future research should focus on optimizing the long‐term stability and reusability of FeNP‐CC through targeted surface functionalization. Additionally, conducting a comprehensive life cycle assessment (LCA) and cost‐benefit analysis will be pivotal to validating the industrial scalability, economic feasibility, and environmental footprint of this promising green nanobiosorbent for sustainable water treatment technologies.

## Author Contributions

**Neethu Cyril**: conceptualization, investigation, writing – original draft, supervision. **James Baben George**: conceptualization, data curation, writing – original draft. **Sylas** **Variyattel Paulose**: conceptualization, supervision. **Laigi Joseph**: investigation, writing – review and editing.

## Funding

These authors received no specific funding for this work.

## Conflicts of Interest

The authors declare that they have no known competing financial interests or personal relationships that could have appeared to influence the work reported in this paper.

## Supporting information



The supplementary file contains detailed information on the qualitative phytochemical screening of *Canavalia cathartica* seed extract, calibration curves for the antioxidant assays performed, and the MS/MS fragmentation pattern confirming the presence of L‐canavanine.
**Supporting File 1**: cbdv71847‐sup‐0001‐SuppMat.docx.

## Data Availability

The data that support the findings of this study are available from the corresponding author upon reasonable request.
