# Supplementary material for: Bioengineered Iron Nanoparticles From Canavalia cathartica Seeds: A Promising Nanobiosorbent for Cr (VI) Remediation
Source: Chem Biodivers. 2026 Oct 20;23(10):e71847. doi: 10.1002/cbdv.71847 (PMC13684051; doi:10.1002/cbdv.71847)
Supplement: Supplementary file 1 [file CBDV-23-e71847-s001.docx]

**Reference to Voucher Specimen of *Canavalia cathartica***

**Qualitative Phytochemical Analysis**

1. Alkaloids: *Dragendorff’s Reagent Test*

To detect the presence of alkaloids, 1 mL of the aqueous plant extract was mixed with 10% acetic acid prepared in ethanol. Subsequently, a few drops of Dragendorff’s reagent were added to the mixture. The formation of a reddish-orange precipitate was taken as a positive indication of alkaloids.

2. Flavonoids: *Alkaline Reagent Test*

To test for flavonoids, 1 mL of the aqueous extract was treated with a few drops of 10% sodium hydroxide (NaOH) solution in a test tube. The immediate appearance of a deep yellow colouration suggests the presence of flavonoids. This colouration is due to the formation of flavonoid sodium salts in alkaline medium. The yellow colour disappearing upon the addition of dilute hydrochloric acid (HCl) further confirms the presence of flavonoids, as the flavonoid salts revert to their original, less coloured acidic form.

3. Saponins: *Froth Test*

For the qualitative detection of saponins, 1 mL of the extract was boiled with 5 mL of distilled water in a test tube. After boiling, the solution was further diluted with 3 mL of distilled water and shaken vigorously. The formation of stable and persistent froth, especially upon gentle warming, was interpreted as a positive result for saponins.

4. Proteins: *Ninhydrin Test*

To determine the presence of proteins or free amino acids, 2 mL of the plant extract was mixed with a few drops of 2% ninhydrin solution. The mixture was thoroughly shaken and then heated in a water bath for approximately 5 minutes. The development of a blue or violet coloration indicated the presence of proteins or amino compounds.

5. Cardiac Glycosides: *Keller–Killiani Test*

To detect cardiac glycosides, particularly steroidal aglycones, 2 mL of the plant extract was mixed with 2 mL of glacial acetic acid containing two drops of 2% ferric chloride (FeCl₃). Carefully, concentrated sulfuric acid (H₂SO₄) was added along the sides of the test tube without disturbing the mixture. The formation of a brown ring at the interface of the two layers was considered a positive indication of the presence of cardiac glycosides.

6. Tannins: *Lead Acetate Test*

The presence of tannins was assessed by adding a few drops of 10% lead acetate solution to 1 mL of the aqueous extract. The appearance of a yellow precipitate indicated a positive test for tannins.

**Qualitative Phytochemical Analysis**

Determination of Total Phenolic Content

The total phenolic content of the aqueous extract of *C. cathartica* was estimated by Folin-Ciocalteu method (Singleton et al. 1999). Total phenolic assay was conducted by mixing 500µL of the extract with 2 mL of 10% Folin reagent and finally neutralized with 4 mL 7.5% sodium carbonate solution. The mixture was incubated at room temperature for 30 minutes with shaking at intervals and the absorbance was measured at 765 nm. Each concentration was analyzed in triplicate A calibration curve was generated using gallic acid as the standard ((y= 0.01122x; R²=0.9991, (Figure S1) and the results were expressed as milligrams of gallic acid equivalents (GAE) per gram of dry sample.

2.3.2 Determination of FRAP activity

The antioxidant potential of the aqueous seed extract was assessed using the Ferric Reducing Antioxidant Power (FRAP) assay, which quantifies the ability of antioxidants to reduce ferric (Fe^3+^) to ferrous (Fe^2+^) ions. Upon reduction, Fe^2+^ forms a stable and intensely blue-coloured complex with, exhibiting a strong absorbance maximum at 595 nm, indicative of effective electron donation by antioxidants. FRAP reagent was freshly prepared by mixing 25 mL of acetate buffer, 2.5 mL of ferric chloride (FeCl_3_), and 2.5 mL of 2,4,6-tri(2-pyridyl)-s-triazine (TPTZ) solution. Sample solutions at varying concentrations (0–50 μg/mL) were made up to 1 mL using the FRAP reagent. The mixtures were then incubated at 37^0^C for 15 minutes to allow complete complexation. The absorbance was subsequently measured at 595 nm using a UV-Visible spectrophotometer. Each concentration was analyzed in triplicate The effective concentration (EC 50) of the extracts—the concentration required to achieve 50% of maximum reducing power—was calculated relative to Vitamin E, which served as a positive control (Figure S2). To quantify the Fe^2+^ generated, a standard calibration curve was constructed using ferrous sulphate having a linear regression equation y = 0.00314x + 0.00153, R^2^ = 0.9947 where y is the absorbance at 593 nm and x is the concentration of ferrous sulphate solution, (Figure S3) and the FRAP value was expressed as mM Fe²⁺ per µg of extract.

$$\% Increase in FRAP activity= \frac{Absorbance of sample-Absorbance of control}{Absorbance of control} \times100$$

2.3.2 Determination of Total Antioxidant Capacity

This assay is based on the reduction of Mo (VI) to Mo (V) by the extract resulting in the formation of green coloured phosphomolybdeneum complex at the acidic pH (Prieto et al. 1999). 50 mL reagent solution was prepared by mixing 0.247 g ammonium molybdate and 0.168 g of sodium dihydrogen phosphate with 1.6 mL of 0.6 M sulphuric acid. 50 μg of *C. cathartica* extract was added to 1 mL of reagent solution and incubated at 95^0^C for 90 minutes. After the mixture was cooled to room temperature, absorbance was taken at 695 nm against the blank. Each concentration was analyzed in triplicate. Trolox was used as the standard. The total antioxidant capacity of extract was determined by using a linear regression equation of calibration curve for trolox, y = 0.0152 x, R^2^ = 0.9846 where y is the absorbance at 695 nm and x is the concentration of trolox in µg/mL (Figure S4) and the total antioxidant capacity was expressed as mg/g equivalents of trolox.

**Figure S1.** Calibration curve of total phenolic content

**Figure S2.** Calibration curve for FRAP assay

**Figure S3.** Calibration curve of ferrous sulphate heptahydrate

**Figure S4.** Calibration curve of trolox for TAC assay

**Figure S5.** The MS spectrum of base peak :177

**Figure S6.** The MS/ MS fragmentation pattern of product ion scan precursor:177
